# Vaccine-enhanced disease: case studies and ethical implications for research and public health

**DOI:** 10.12688/wellcomeopenres.16849.1

**Published:** 2021-06-16

**Authors:** Euzebiusz Jamrozik, George Heriot, Susan Bull, Michael Parker, Oxford-Johns Hopkins Global Infectious Disease Ethics (GLIDE)

**Affiliations:** 1 The Ethox Centre & Wellcome Centre for Ethics and Humanities, University of Oxford, Oxford, UK; 2 Monash Bioethics Centre, Monash University, Melbourne, Australia; 3 Department of Medicine, Royal Melbourne Hospital, University of Melbourne, Melbourne, Australia

**Keywords:** Vaccine, ethics, uncertainty, risk, respiratory syncytial virus, measles, dengue, Staphylococcus aureus.

## Abstract

Vaccination is a cornerstone of global public health. Although licensed vaccines are generally extremely safe, both experimental and licensed vaccines are sometimes associated with rare serious adverse events. Vaccine-enhanced disease (VED) is a type of adverse event in which disease severity is increased when a person who has received the vaccine is later infected with the relevant pathogen. VED can occur during research with experimental vaccines and/or after vaccine licensure, sometimes months or years after a person receives a vaccine. Both research ethics and public health policy should therefore address the potential for disease enhancement. Significant VED has occurred in humans with vaccines for four pathogens: measles virus, respiratory syncytial virus, Staphylococcus aureus, and dengue virus; it has also occurred in veterinary research and in animal studies of human coronavirus vaccines. Some of the immunological mechanisms involved are now well-described, but VED overall remains difficult to predict with certainty, including during public health implementation of novel vaccines. This paper summarises the four known cases in humans and explores key ethical implications. Although rare, VED has important ethical implications because it can cause serious harm, including death, and such harms can undermine vaccine confidence more generally – leading to larger public health problems. The possibility of VED remains an important challenge for current and future vaccine development and deployment. We conclude this paper by summarising approaches to the reduction of risks and uncertainties related to VED, and the promotion of public trust in vaccines.

## Disclaimer

The views expressed in this article are those of the authors. Publication in Wellcome Open Research does not imply endorsement by Wellcome.

## Introduction

Vaccination is one of the greatest achievements of public health and infectious disease research. Standard licensed vaccines are generally extremely safe, and typically the direct individual benefits of vaccination significantly outweigh any risks or side effects. In addition, the immunity of vaccinated individuals provides further public health benefits by indirect protection of others, collectively creating population (herd) immunity. Nevertheless, rare serious adverse effects do occur with experimental vaccines, sometimes halting vaccine development, and have also occurred with licensed vaccines, in exceptional cases leading to restriction or withdrawal of public health use.

Vaccine-enhanced disease (VED) is a distinct type of infection-related adverse event that occurs when disease severity is increased following exposure to the relevant pathogen after vaccination ^1^. A key difference between VED and other adverse events is that it is contingent on post-vaccination infection with the relevant pathogen, whereas other types of adverse events are caused by the vaccine itself (including in rare cases where the microbes in live-attenuated vaccines themselves cause disease) or direct immune responses to the vaccine. Other authors have provided guidance for the detection of a correlation between vaccination and increased disease severity and the confirmation of likely VED with additional evidence supporting a causal link between vaccination and enhanced disease ^1^.

Significant VED has occurred for at least four human vaccines, although the terminology used to describe disease enhancement has been different in each case ( Table 1). This article reviews these four case studies of VED in humans over the last six decades and explores the ethical implications for vaccine research and public health policy. The phenomenon of VED has recently received greater public attention because of concerns that COVID-19 vaccines might cause VED, due to disease enhancement observed in animal studies of experimental vaccines for other human coronaviruses ^2, 3^.

**Table 1.** Cases of vaccine-enhanced disease and relevant outcomes.

Pathogen	Term for specific VED	Time period	VED identified during research or implementation	Presumed mechanism(s)	Outcome
Measles	‘atypical measles’	1960s	Implementation	Th2-biased immune response, low avidity antibody	Licensed vaccine withdrawn
RSV	‘enhanced respiratory disease’	1960s	Research	Th2-biased immune response, low avidity antibody	Vaccine not licenced, two deaths
*Staphylococcus* *aureus*	N/A	2010s	Research	Unknown	Vaccine not licensed, 12 deaths
Dengue	‘secondary-like’ disease	2016- present	Research & implementation	Antibody-dependent enhancement via Fcγ receptor	Vaccine use restricted, uncertain number of deaths, public controversy, effects on vaccine confidence

Reference for mechanisms of measles, RSV, dengue enhancement ^7^. RSV, respiratory syncytial virus; VED, vaccine-enhanced disease.

The ethical implications of VED for research and public policy require careful consideration because, although rare, VED can cause serious harm (including death) and has the potential to undermine public trust in vaccines ^4, 5^. Previous cases of VED have led to changes in research and public health practice ^5, 6^, yet the topic has not been widely discussed from an ethical perspective. We argue for careful and transparent evaluation of, and communication regarding, the risks and uncertainties regarding VED for novel vaccines. Where residual risks and uncertainties remain, these should be weighed against potential benefits and monitored during follow-up of recipients of novel vaccines.

## Case studies

The four case studies below highlight the issues and challenges that arose in VED associated with measles virus, respiratory syncytial virus (RSV), *Staphylococcus aureus*, and dengue virus. Many of the points raised may also be relevant to vaccine development for other pathogens, and to some related types of adverse effects ^7, 8^. Some experimental vaccines, for example, have been associated with increased (as opposed to decreased) risks of infection without enhancement of disease (i.e., infection after vaccination is more likely, but the clinical severity of the resulting infection is not more severe than average). At least one human influenza vaccine has been associated with an increased rate of influenza diagnosis but no increased risk of hospitalisation ^9, 10^. Similarly, one experimental HIV vaccine was associated with an increased risk of infection, with no evidence that the natural history of the resulting HIV infections and/or disease outcomes were otherwise altered ^11^. In animal studies, VED has also been noted with veterinary vaccines (e.g., for animal coronaviruses) and animal models intended for human vaccine development (e.g., for human coronaviruses, influenza, and West Nile virus) ^7^. This paper focuses only on cases where there is convincing evidence that vaccines have led to disease enhancement in humans, as opposed to evidence of an association between vaccination and more severe disease without confirmatory evidence of a causal relationship, or evidence of an increased frequency of infection but not increased severity of disease, (e.g., the influenza and HIV vaccines described above). Following these case studies, and against the background of the understanding they provide of VED, we then analyse the ethical issues presented by the development and use of vaccines.

### Case study one: Measles virus

Measles is a highly transmissible virus that is particularly harmful to infants or in those who are first infected in adulthood, as well as to those with comorbidities. Although modern live measles vaccines are highly safe and effective, measles still causes around 100,000 deaths per year, primarily in low-income communities where access to vaccination is poor. Despite the safety of current vaccines, outbreaks of measles have increased in some countries due to reduced vaccination rates partly attributable to vaccine hesitancy ^12^.

From 1961–1967, a licensed measles vaccine created with formalin-inactivated (killed) virus, was associated with a type of VED referred to as ‘atypical measles’ ^13, 14^. Vaccinated individuals with atypical measles develop a severe clinical syndrome with rash and fever and a higher rate of lung involvement (pneumonitis) than usual cases of measles and, in some cases, liver dysfunction and abdominal pain ^13, 14^. By the time the enhanced disease syndrome was characterised, over 1.8 million people, mostly children, had received the vaccine ^13^. The vaccine was withdrawn from public health use due to VED in 1967 ^14^. To our knowledge, there are no estimates of the total number of atypical measles cases due to the vaccine, although sporadic cases are still being reported ^15^. To reduce subsequent risk of atypical measles, individuals who had received the withdrawn vaccine were re-vaccinated with an alternative (live-attenuated) vaccine ^13^.

The initial phase III trial of the formalin-inactivated measles vaccine demonstrated 81% efficacy at 0–3 months after vaccination; however, vaccine efficacy waned over time. At 13 months, efficacy had reduced to 65% ^16^. In addition to waning of immunity, interpretation of vaccine efficacy was complicated by changing patterns of community measles transmission. In some cases, vaccinated children experienced apparently normal measles disease post-vaccination (i.e., neither attenuated nor enhanced, likely due to vaccine failure). In others, it was hypothesised that “asymptomatic infections with wild measles viruses may have served to boost some with low and borderline [antibody] titers” ^16^ (i.e., post-vaccination infection with the wild-type virus was attenuated as a result of vaccination, and the combination of vaccine-derived and post-infection immunity could provide augmented and/or durable protection against subsequent infection) ^16^. Early aggregate vaccine efficacy estimates in the study population may have obscured some short-term cases of VED and the short duration of the initial phase III trial meant that longer-term risks of VED due to waning of post-vaccination immunity over time were not detected until public health use of the vaccine, well after trial completion and vaccine approval ^13^.

### Case study two: Respiratory syncytial virus

Respiratory syncytial virus (RSV) is a ubiquitous virus and a major cause of hospitalisation of young children worldwide, causing up to around 200,000 deaths in children under five per year, primarily in low-income communities ^17^. The first infection with RSV is usually the most severe, typically causing the clinical syndrome of bronchiolitis (lung inflammation and congestion). In more severe cases, RSV causes respiratory failure and death. Naturally acquired immunity to RSV wanes over time, and individuals are commonly re-infected many times throughout their lives despite prior infection. It is increasingly recognised that RSV results in significant mortality among older adults, despite numerous prior infections and some degree of immunity ^18^.

There is currently no licensed vaccine for RSV (although at least one is in phase III clinical trials) in part because RSV vaccine research was impeded by the occurrence of a high-profile case of VED. In 1966, an experimental RSV vaccine studied in toddlers resulted in many severe cases of what was termed ‘enhanced respiratory disease’ (i.e., more severe lung inflammation and respiratory failure) among child participants subsequently exposed to wild-type RSV, including the death of two toddlers. This led to extreme caution regarding further RSV vaccine research ^19^.

Thorough investigation of human cases and preservation of stocks of the 1966 vaccine informed the development of an animal model that closely replicates RSV VED ^20^. This has provided opportunities for research on the pathogenesis of RSV VED as well as the prospective testing of future RSV vaccines against the model to ensure that they do not cause similar pathology. Although animal models have identified VED in vaccines which, as a result, did not proceed to human trials ^8^, earlier animal models (before RSV VED was identified) had failed to detect this risk. This highlights the limitations of animal models in eliminating the risk of VED (among other adverse effects).

### Case study three: Staphylococcus aureus

*Staphylococcus aureus* is a common species of bacteria found on the skin and upper respiratory tract. Around 30% of the world’s population carries *S. aureus*, usually without symptoms, yet it causes up to 600 total infections and 30–40 invasive infections, (i.e., with septicaemia, abscess, or vital organ involvement) per 100,000 person-years ^21– 23^, with a particularly high incidence noted among hospitalised patients, resulting in significant morbidity and mortality ^24, 25^. Early reinfection is common after one episode of clinical *S. aureus* disease (particularly in patients with risk factors for invasion) ^26^ and immunity to *S. aureus* is incompletely understood as antibodies to the bacteria are both ubiquitous and insufficient to prevent reinfection ^27^. Moreover, the prevalence of resistant *S. aureus* is increasing worldwide, and WHO has identified methicillin-resistant *S. aureus* as a high priority resistant pathogen for which better control methods are urgently needed.

Of the 15 antigenic targets for a *S. aureus* vaccine identified in pre-clinical studies, none have resulted in effective human vaccine targets, despite three candidate vaccines reaching phase IIB/III trials ^28– 31^. One of these candidates (non-adjuvated IsdB) was associated with a five-fold increase in the mortality of patients with *S. aureus* infection occurring after cardiothoracic surgery (from 4% to 26%). This apparent VED was not seen in pre-clinical mouse challenge studies of the IsdB vaccine or phase I/II trials of healthy volunteers ^32, 33^. Post-hoc analyses of the trial subjects experiencing *S. aureus* infection suggested that individuals with particular pre-vaccination cytokine signatures (low serum IL-2 and/or IL17a) experienced high infection-related mortality after vaccination (but not after placebo administration), but the mechanisms of this case of VED remain poorly understood.

A subsequent large phase III trial of another candidate vaccine aimed at enhancing opsonophagocytic killing of *S. aureus* was not associated with VED in high-risk patients, despite very similar preclinical observations and lack of overall efficacy ^29, 34^. This serves to underscore the unpredictability of VED in late-stage trials, especially for pathogens with complicated and incomplete human immunity.

### Case study 4: Dengue virus

Dengue is a vector-borne arboviral disease caused by four related strains of dengue virus. Dengue causes millions of cases per year in endemic areas, and increasing numbers of people are at risk ^35^. A key feature of dengue is that while first infection is often mild or asymptomatic, second infection (with a different strain to the first infection) is the most likely to be severe, especially if the antibody response to first infection has waned to a certain level, potentiating antibody-dependent disease enhancement ^36^. Severe dengue occurs in approximately 2–5% of secondary infections and sometimes results in death ^37^. Third and subsequent infections (with any strain) are typically mild or asymptomatic.

A 2015 study of an experimental tetravalent dengue vaccine (known as CYD-TDV) which enrolled children in endemic areas revealed that the vaccine was, overall, associated with a 60% reduction in symptomatic dengue. However, in some younger children, the vaccine was associated with increased risks; for example, vaccinated children aged 2–5 in the Asia-Pacific arm of the trial were 7.45 times more likely to be hospitalized with severe dengue than those in the control group ^38^. At the time, it was thought that the most likely reason for higher risks in some children was that the live vaccine primed the immune system in a similar way to a first dengue infection among those who had never been infected (seronegative children). When these individuals then had a naturally-acquired wild-type infection after being vaccinated, this resulted in ‘secondary-like’ VED (i.e., more likely to be severe). Not every person in an endemic area is exposed to dengue every year, and it can take several years before children are infected for the first time, i.e., before ‘seronegative’ individuals become ‘seropositive’. In older age groups in endemic areas, the majority are seropositive – and seropositive people (particularly those who have only been infected once before) appear to benefit from CYD-TDV vaccination.

In contrast to the cases discussed above, there is therefore good reason to think that CYD-TDV could provide net public health benefits, either by vaccinating only seropositive individuals or by vaccinating highly seropositive populations (although this strategy exposes a minority of individuals to a risk of VED). Public health modelling suggested that widespread use of CYD-TDV in populations with high proportion of seropositive individuals could reduce the burden of dengue disease by 10–40% over 10 years ^1^. The vaccine was approved by WHO’s Strategic Advisory Group of Experts (SAGE) for use in children over the age of nine in endemic areas where the proportion of seropositive individuals was greater than 70%. The vaccine was initially rolled out without routine pre-vaccination serological testing (due to economic and technical constraints ^6^) but with a plan to seek further data regarding the elevated risk in seronegative individuals ^6^.

In contrast to the hypothesis that dengue VED was merely akin to secondary dengue infection, researchers not involved in the development of the vaccine estimated that CYD-TDV VED was up to 3.5 times more (likely to be) severe than usual secondary dengue infection ^39^. These authors recommended in 2016 that CYD-TDV vaccination be restricted to seropositive individuals “regardless of inconvenience or cost.” ^39, 40^. Later, controversy ensued because after vaccination campaigns had begun, results confirming the risk of VED among seronegative individuals were published. In the Philippines, where over 800,000 children had been vaccinated, the controversy resulted in political uproar and a decline in confidence regarding vaccines in general ^4, 41^.

Subsequently, SAGE convened a working group including an ethicist. The group proposed a change of policy to restrict the use of CYD-TDV to seropositive individuals, in whom there was clear evidence that the vaccine was safe and effective ^6^. However, this resulted in significantly less public health use of the vaccine, because there is still no cost-effective serology testing strategy that could be used routinely to guide dengue vaccination strategies in endemic areas. It is likely that many thousands of seronegative individuals in endemic areas were vaccinated with CYD-TDV prior to the revised policy, and these individuals face a risk of VED if infected after vaccination. The true burden of CYD-TDV VED is difficult to estimate because seronegative individuals were not identified at the time of vaccination and because although it is known that the risk of VED persists for several years, the longer-term risks are poorly characterised.

## Ethical implications

 Although rare, VED has been associated with significant harms and has often been difficult to predict with a high degree of certainty. There is, therefore, a strong ethical rationale for measures to minimise risk and uncertainty regarding VED for novel vaccines, as well as for transparent communication regarding any residual risks and uncertainties. This is important not only to protect participants in vaccine trials and early recipients of newly approved vaccines, but also to promote public trust in vaccines. In some cases public trust has been undermined by the occurrence of VED and/or a lack of transparent public communication regarding such risks ^41, 42^. Below, we discuss relevant aspects of risk and uncertainty in more detail, including in the context of COVID-19 vaccine research and human challenge studies, before highlighting ethical implications for policymaking related to the public health implementation of vaccines.

### Risk and uncertainty

Consensus standards in research ethics require, among other things, that risks to participants are carefully evaluated, minimised, and that residual risks are justified by the social value of the research. This requires comprehensive, rigorous, and systematic evaluation of the anticipated risks, burdens, and benefits of proposed research. Risks are sometimes distinguished from uncertainties by defining risks as harmful outcomes with a known magnitude and probability and uncertainties as potential outcomes with unknown probabilities and/or magnitudes. This sharp distinction between known and unknown outcomes obscures the frequent occurrence of situations in which estimates of the probability or magnitude of an outcome are more or less certain (reflected, for example, by narrower or wider confidence intervals around a risk estimate), ranging from zero certainty (strict uncertainty) to high certainty. In some cases, researchers may be aware of the *possibility* of particular outcomes without being able to characterise the *probability* of these outcomes with any certainty. In other cases, even the possibility of a particular outcome is unknown (or not considered) prior to its occurrence (situations of ignorance or so-called ‘unknown unknowns’) ^43^. All such situations are captured by various uses of the term ‘uncertainty’. Early phase and first-in-human clinical research inevitably involves significant uncertainty with respect to both benefits and harms, meaning that both good and bad outcomes are sometimes poorly matched to those expected based on prior data ^44^. 

The case studies above feature both risks and uncertainties. For example, at the time of licensure of the CYD dengue vaccine, there were risks because a phase III trial had demonstrated a harm signal and the potential mechanism was well-described. There were also uncertainties regarding the probability and magnitude of the risk of VED in certain individuals and groups. In other cases where no risks are identified in earlier (animal or human) studies, the potential for VED during vaccine development and after licensure is highly uncertain. For example, in the other cases above, VED was not expected to occur during the phase III *S. aureus* vaccine trial in surgical patients who would face significant harms from such an enhanced disease, nor among children given the experimental RSV vaccine, nor during public health use of the measles vaccine. 

The primary role of vaccine development processes, which involve gathering more data on safety, immunology, and efficacy, is to reduce or resolve uncertainties. Although safety is a key focus of preclinical and early clinical research, most measures of safety become more certain in later-phase research. However, because VED does not occur until a person is exposed to a subsequent infection, which might occur only in late phase research and/or after a considerable time has passed since vaccination. VED can therefore remain a safety concern and area of uncertainty during phase III (efficacy) testing, which necessarily involves participants being exposed to infection with the pathogen in question ^45^. Safety concerns about VED may also remain relevant (or be first identified) in post-licensure surveillance, as demonstrated in the case studies of measles and dengue.

Minimising risks to vaccine trial participants necessarily involves trade-offs between the interests of trial participants and considerations related to scientific validity and/or efficiency, both of which are ethically salient to producing public health benefits associated with novel vaccines ^44^. A key focus of risk minimisation is the reduction of the probability of serious or irreversible harms – which are a potential consequence of VED. Phase III trials may seek to recruit individuals who face a high *probability* of the infection in question, because this may optimise scientific validity/generalisability, produce results more rapidly, and because individuals at risk may be independently motivated to participate. In some cases, however, VED may be particularly harmful in groups where the *outcome* of enhanced disease would be particularly severe (e.g., thoracic surgical patients in the *S. aureus* case above). It may also be infeasible to reduce the risk of VED in remaining participants after it is observed (in some participants) during the trial – although the measles case illustrates that where other safe vaccines are available, re-vaccination with an alternative vaccine for the same pathogen may mitigate future VED risks.

Both for reasons of continued uncertainty and high magnitude of potential harm, phase III research should arguably involve particularly vigilant monitoring for VED. Where there is concern regarding potential VED, optimal risk minimisation may include particularly careful participant selection criteria and the exclusion of children where feasible. For example, because individuals are reinfected with RSV many times in a lifetime, it has been feasible to exclude children in the initial trials of novel RSV vaccines conducted since the case of VED described above, followed by cautious inclusion of children in subsequent studies once safety is demonstrated in adults.

Follow-up of all novel vaccine trial participants should arguably be of a long duration to detect rare late adverse effects including the potential for late VED. Exposure to infection may occur many months or years after the end of a trial and in some cases the risk of VED may increase with delayed infection (e.g., where the waning of post-vaccination immune response alters the risk of VED) ^36^. Waning immunity may explain why the inactivated measles vaccine showed overall population protection during initial trials but was eventually shown to be associated with a significant risk of VED during public health implementation in the general community. Similarly, variations in the local incidence of infection during and after a phase III trial may alter the ability to detect VED. The incidence of dengue was highly variable during and after the initial multi-country CYD-TDV phase III study ^46^, which may have contributed to variable efficacy findings in later years of the trial, including the harm signal suggestive of VED.

### Implications for COVID-19 vaccines

Experimental vaccines for SARS-CoV and MERS-CoV have been associated with evidence of VED in animal models, which raised concerns regarding the potential for VED with COVID-19 vaccines ^8^. A March 2020 expert meeting regarding the potential for COVID-19 VED proposed several measures to reduce the risks of VED including (i) vigilant investigation of vaccine immune responses for those previously associated with VED; (ii) the use of animal challenge models that adequately mimic human disease, ideally including adequate time delay between the vaccination of animals and infection challenge; (iii) consideration of antibody transfer from phase I/II vaccine trial human participants to animal challenge study subjects to test for VED; and (iv) longer follow-up of trial participants (to monitor for the increased disease severity upon exposure to the infection in question) ^8^. Even with these resource-intensive measures in place, it is not possible to exclude the risk of VED altogether – in other words there will often be residual uncertainty – and trials of COVID-19 vaccines have involved informing volunteers regarding the risks and uncertainties related to VED ^47^. Fortunately, no cases of COVID-19 VED have been documented in phase I-III trial participants ^1^, yet this does not exclude the possibility of delayed disease enhancement in the context of waning immune responses. Clinicians and public health agencies should therefore thoroughly investigate any unusually severe cases of COVID-19 in vaccine recipients, ideally using recently proposed standardized criteria for the detection and confirmation of VED ^1^.

### Risks of vaccine-enhanced disease in human challenge studies versus standard vaccine trials

Human challenge studies involve exposing research participants to infection under controlled conditions and are often used to estimate the efficacy of experimental vaccines before proceeding to larger trials ^48^. Phase III vaccine challenge trials typically involve around 100 rigorously screened young healthy adults, whereas standard phase III trials involve tens of thousands of individuals and often a wider age range of participants. Challenge trials permit especially close monitoring of participants for the duration of their infection. On the one hand, challenge studies are ethically sensitive partly because they involve the intentional exposure of participants to risk of infection, potentially including VED (although VED has not occurred in challenge studies to date). Since challenge studies are of short duration, they might detect short-term VED (such as those occurring with RSV and *S. aureus* above) but not delayed VED events (such as those that might occur during waning immunity). On the other hand, were VED to occur in a challenge study, far fewer participants would be exposed to such harms than if it were to occur in a standard phase III trial, participants would be more likely to have immediate access to treatment for any enhanced disease and the evidence of VED would prevent much larger numbers of people being exposed to that vaccine in a standard phase III trial. These competing considerations mean that there may be difficult ethical judgements to be made as to whether challenge studies or field trials are the optimal first method of testing vaccines for diseases where VED is a concern. However, because standard trials involve exposing many thousands more individuals to an experimental vaccine than challenge studies, cumulative risks of VED may be far higher in standard trials ^48, 49^. In particular, where there are many vaccine candidates, prioritising these candidates in challenge studies before proceeding to larger standard trials with only the most promising vaccines may reduce overall aggregate risks (including those related to VED) ^49, 50^.

### Public health implementation

Vaccines typically provide both individual benefit (i.e., net risk reduction) as well as population level benefits (the sum of individual direct benefits plus indirect protection of others, with the combined effect of the latter constituting population ‘herd’ immunity). The occurrence of VED may alter the balance of risks and benefits of vaccine implementation in at least two ways. First, a particular sub-population may face a net risk from vaccination due to VED (despite overall population benefits). Importantly, in such cases, aggregate statistics from a phase III study may demonstrate that a vaccine is efficacious (i.e., beneficial) at the population level because the majority of individuals are protected even if a minority experience VED (see dengue and measles cases above). Researchers should therefore take care to investigate whether overall efficacy estimates conceal cases of VED as well as how benefits and harms associated with vaccination are distributed in the population. Where a vaccine is licensed despite a risk of VED in a minority of vaccine recipients, relevant public health implementation policies should be considered with the utmost care – and public health agencies should engage with sub-populations who might be placed at greater risk. Such engagement and policymaking should include transparent disclosure of relevant risks and uncertainties, the development of fair procedures for the identification of higher risk individuals (if possible), attention to how risks can be minimised, and provision for compensation for serious harms if these occur.

Where the sub-population at risk of VED due to a licensed vaccine is readily identifiable, it is important to consider whether they should be excluded from mass vaccination, although this may undermine the overall efficiency and/or cost-effectiveness of vaccine programs, especially if identifying higher risk individuals requires testing (e.g., of serostatus in the case of dengue) ^6^. In addition to making policy based on the overall balance of potential health benefits and risks at the population level, the distribution of risk should also be considered – for example, it may be ethically problematic to impose risks on already marginalised sub-populations or those who cannot provide consent (e.g., children).

Where a sub-population is not readily identifiable, it may sometimes be more appropriate to make a vaccine that offers both significant potential benefits and significant risks (e.g., of VED) a matter of informed consent based on individual doctor-patient discussion of risks and benefits rather than as part of a mass vaccination strategy where the default is to be vaccinated. This informed consent approach is often employed for pre-travel use of the relatively risky but protective vaccines for Japanese encephalitis and yellow fever, for example.

Second, VED can alter the balance of risks and potential benefits of vaccination over time, whether it affects a sub-population or all vaccine recipients. For example, this might occur where a vaccine provides some degree of individual protection against disease in the short-term, but those who are exposed after a longer period of time experience not only less protection but also a higher risk of VED (which is one potential explanation for the epidemiology of atypical measles). In the case of dengue, mass CYD-TDV vaccination of high seroprevalence populations might provide overall population health benefits in the short-to-medium term despite exposing a minority to the risks of VED, but in the long term continued mass vaccination and reduced incidence of dengue might result in increasingly large numbers of seronegative people experiencing VED if/when dengue epidemics recur ^51^.

### Maintaining public confidence in vaccines

It is widely acknowledged that trust in licensed vaccines, vaccine research, and public health agencies is essential for the ethical acceptability and long-term success of public health programs – and vaccine hesitancy is considered a ‘top 10’ threat to global health ^50^. Since VED and other rare but serious harms from vaccination have been associated with declines in public trust, policymakers should prepare for such outcomes. Public health agencies have a responsibility to develop evidence-based approaches to engage with target populations about the benefits, risks, and uncertainties of vaccine programmes (including VED where relevant) and to respond to their perspectives, concerns, and expectations. Engagement with relevant populations regarding vaccination should include careful transparent disclosure of the potential for VED where this is reasonable concern ^5, 6, 42^. Other policies that might help to promote trust include commitments to undertake vigilant surveillance for vaccine side-effects, especially those that involve significant harm, and to provide appropriate compensation if any vaccine-related harms occur. Where a vaccine is planned for global use, it may also be appropriate for high-income countries or vaccine development sponsors to fund compensation programmes for people in low-income countries who may not otherwise have access to such remediation, especially where global vaccination programs seek to achieve collective aims such as disease eradication ^52^.

## Conclusions

Vaccination is a cornerstone of global public health, and novel vaccines are developed in order to produce additional health benefits. Although rare, the potential for disease enhancement during vaccine research or public health use of novel vaccines remains a key source of uncertainty and potential risk, and cases of VED have resulted in serious harm as well as declining public trust in vaccines. Vaccine development and implementation should therefore involve measures to reduce risks and uncertainty during vaccine research, transparent communication regarding residual risks and uncertainties, and compensation for any research-related harms, including VED. At the level of public health policy, it should be acknowledged that novel vaccines sometimes produce unexpected harm, including VED, and ongoing public engagement programs should be carefully designed to maintain and promote public trust in vaccination, especially where there are any concerns regarding potential disease enhancement.

## Data availability

No data are associated with this article.
