# Supplementary material for: Vaccine-enhanced disease: case studies and ethical implications for research and public health
Source: Wellcome Open Res. 2021 Jun 16;6:154. [Version 1] doi: 10.12688/wellcomeopenres.16849.1 (PMC8250497; doi:10.12688/wellcomeopenres.16849.1)
Supplement: Peer review history

# Peer review: Reviewer response for version 1

**DOI:** 10.21956/wellcomeopenres.18584.r44512

**Published:** 2021-07-02

**Authors:** Peter Collignon

**Affiliations:** 1 Medical School, Australian National University, Canberra, ACT, Australia

This is a well written and important paper for anyone to read when examining the issue of Vaccine safety. 

 The conclusion gives a very good summary and maybe some more of it in part could be incorporated into the Abstract as often the abstract is all readers read.

 The study by Fowler *et al.* should be separately referenced when they mention the increased mortality rates in the group receiving the Staph aureus vaccine, so the reader can more easily find the appropriate reference.

Does the article adequately reference differing views and opinions?

Yes

Are all factual statements correct, and are statements and arguments made adequately supported by citations?

Yes

Is the Open Letter written in accessible language?

Yes

Where applicable, are recommendations and next steps explained clearly for others to follow?

Yes

Is the rationale for the Open Letter provided in sufficient detail?

Yes

Reviewer Expertise:

Infectious diseases , epidemiology, microbiology and Infection control and Prevention

I confirm that I have read this submission and believe that I have an appropriate level of expertise to confirm that it is of an acceptable scientific standard.

# Peer review: Reviewer response for version 1

**DOI:** 10.21956/wellcomeopenres.18584.r44514

**Published:** 2021-07-01

**Authors:** Mirko Ancillotti

**Affiliations:** 1 Department of Public Health and Caring Sciences, Centre for Research Ethics & Bioethics, Uppsala University, Uppsala, Sweden

This article explores the ethical implications of vaccine-enhanced disease (VDE). The article is clear and of sure interest. I have definitely appreciated the description and analysis of the four cases, which provide evidence-based grounds for the normative discussion. I recommend the publication of this article.

 I have a few minor comments that I would like to share with the authors (not a condition for publication, however).

 In the Introduction, p.3 of the pdf, it is written: “The ethical implications of VED for research and public policy require careful consideration because, although rare, VED can cause serious harm (including death) and has the potential to undermine public trust in vaccines”. My focus is on the public trust issue, which is repeated multiple times in the article. For instance, at p.8: “Since VED and other rare but serious harms from vaccination have been associated with declines in public trust, policymakers should prepare for such outcomes”.

 It is claimed that VED may affect public trust. To my understanding reading the article, trust appears to be undermined by how VED risks/uncertainties translate into vaccination policy and how this is communicated. The authors may consider it obvious and/or implicit. Nonetheless, statements like “VED can cause serious harm (including death) and has the potential to undermine public trust in vaccines” may suggest that the problem is VED per se *,* in the sense that morbidity and mortality of VED are particularly high and can alter the overall risk/benefit proportion. And given that a continuous assessment of risks and benefits is necessary to strengthen the confidence in immunization programmes, then it would be the impact of VED per se that need to be monitored to ensure public trust. Of course, one does not necessarily exclude the other but the quoted statements (and others in the article) may suggest that VED per se is the problem that may undermine public trust.

 On a minor note, the description of the outcome of dengue vaccine in the table is “Vaccine use restricted, uncertain number of deaths, public controversy, effects on vaccine confidence”. Thus, a set of very different outcomes is considered. While ‘Vaccine use restricted’ and ‘uncertain number of deaths’ are in line with the description of the outcomes offered for the other three cases — the decision about the vaccine license and deaths — ‘public controversy’ and ‘effects on vaccine confidence’ refer to a completely different type of outcomes (essentially, public trust). These trust issues emerged only in response to the dengue vaccine? If yes, the table does not need any modifications. If no, the authors may consider to cancel ‘public controversy’ and ‘uncertain number of deaths’ from the description of dengue vaccine outcome (or add the description of potential consequences on public trust to the others).

Does the article adequately reference differing views and opinions?

Yes

Are all factual statements correct, and are statements and arguments made adequately supported by citations?

Yes

Is the Open Letter written in accessible language?

Yes

Where applicable, are recommendations and next steps explained clearly for others to follow?

Yes

Is the rationale for the Open Letter provided in sufficient detail?

Yes

Reviewer Expertise:

Bioethics

I confirm that I have read this submission and believe that I have an appropriate level of expertise to confirm that it is of an acceptable scientific standard.

# Peer review: Reviewer response for version 1

**DOI:** 10.21956/wellcomeopenres.18584.r44516

**Published:** 2021-06-17

**Authors:** Zohar Lederman

**Affiliations:** 1 Rambam Medical Campus, Haifa, Israel

This article discussed the ethical implications of vaccine-enhanced disease, or when vaccination augments the symptoms and signs of a communicable disease. It is clearly written, and it addresses a novel and pertinent issue in bioethics, with a coherent normative deliberation based on solid empirical ground. In light of these, it should be indexed.

 Several minor comments for consideration (not necessary to address for acceptance):

1. “In other cases where no risks are identified in earlier (animal or human) studies, the potential for VED during vaccine development and after licensure is highly uncertain. For example, in the other cases above, VED was not expected to occur during the phase III S. aureus vaccine trial in surgical patients who would face significant harms from such an enhanced disease, nor among children given the experimental RSV vaccine, nor during public health use of the measles vaccine.”
  I wonder whether ‘highly uncertain’ correlates with ‘not expected’- I would think that highly uncertain means that its effects were simply unknown, while not expected means that based on available knowledge risk was determined to be minimal. In this case, the latter seems to be what the authors want to say.
2. “Follow-up of all novel vaccine trial participants should arguably be of a long duration to detect rare late adverse effects including the potential for late VED”
  I wonder how feasible this is? What is long enough? Otherwise put, what is the threshold for enough certainty in order to license a vaccine? And who determines the threshold? The obvious player may be the WHO, but what about individual communities? Maybe certain communities would prefer higher stakes for potentially higher gains or vice versa?
3. It might be worthwhile to sound a warning about uncertainty delaying scientific progress.   After all, people are dying from infectious diseases, and the more we wait for certainty, the more will die, perhaps unnecessarily.
4. “but in the long term continued mass vaccination and reduced incidence of dengue might result in increasingly large numbers of seronegative people experiencing VED if/when dengue epidemics recur” The article is extremely nuanced, but I wonder whether this consideration may be too nuanced. Worrying about what might happen if we achieve herd immunity against Dengue in terms of VED is like worrying about our planet and its subsistence when we consider developing a cure for cancer. Sure, it’s something to think about, the solution may exist or appear in the future and in any case, this is a tangential consideration in the current context. In other words, it is OK to leave some tangential considerations aside.
5. “Public health agencies have a responsibility to develop evidence-based approaches to engage with target popuations about the benefits, risks, and uncertainties of vaccine programmes (including VED where relevant) and to respond to their perspectives, concerns, and expectations. Engagement with relevant populations regarding vaccination should include careful transparent disclosure of the potential for VED where this is reasonable concern” this sounds wonderful, but you know human psychology makes things much more complex. The manner in which data will be presented (and by whom) or views are gained will affect the outcome of the so-called engagement. If Covid-19 has shown us anything, it is that many folks tend to be conservative, opting to take their chances with a pathogen rather than uncertainty or some rare side effects. I have no solution to this and am not sure how the authors could address this issue, or if in fact, they should in this article.

 To conclude, I guess my greatest concern with this fine piece is the lack of local voices in the decision-making process and the psychology involved in the mainstreaming and application of the scientific and normative complexity addressed here. Simply ‘engaging’ with local communities may not be enough. Public health is at the end of the day political, and- whether we as ethicists want it or not- people will make decisions based on what appeals to them most, rather than what is just or scientifically justified. Again, I think the authors should be allowed not to enter this rabbit hole.

Does the article adequately reference differing views and opinions?

Yes

Are all factual statements correct, and are statements and arguments made adequately supported by citations?

Yes

Is the Open Letter written in accessible language?

Yes

Where applicable, are recommendations and next steps explained clearly for others to follow?

Yes

Is the rationale for the Open Letter provided in sufficient detail?

Yes

Reviewer Expertise:

public health ethics, infectious disease ethics, One Health ethics.

I confirm that I have read this submission and believe that I have an appropriate level of expertise to confirm that it is of an acceptable scientific standard.
